# Legislation

**Published:** 1897-06

**Authors:** 


### LEGISLATION.

*Pennsylvania.* A bill has been introduced appropriating $50,000 for the investigation of contagious and infectious diseases of domestic animals. This bill has been referred to the Appropriation Committee, with strong indorsements and general approval.

A bill to prohibit the importation of diseased cattle into Pennsylvania has just passed first reading in the Senate, and is on the calendar for second reading.

The bill requiring all animals entering the State to replenish our dairies to have been tested and pronounced free from tuberculosis, or to remain at the point of entry sufficient time to allow testing with tuberculin, has passed both Houses and been signed by the Governor, and goes into effect January 1, 1898.

*New York.* The lower branch of the Legislature passed the bill forbidding the sale of horseflesh for food.

The State Board of Health has obtained an appropriation in the annual supply bill of $15,000 for the continuance of inspection of animals for tuberculosis; $7500 for glandered horses, and in the Supplementary Supply Bill $10,000 additional was appropriated for cattle inspection, making in all $25,000 for this work in the Empire State. Later information reaches us that this amount was cut out of the Supply Bill by the Governor.

Of the dozen or more bills placed before the Legislature of the Empire State in which the veterinarian was interested directly or indirectly, every one was lost by defeat or allowed to sleep in the hands of the various committees. No legislation was secured, except that in the way of an appropriation to the State Board of Health for further investigation of bovine tuberculosis.

*National.*—Army legislation looking to the recognition of the veterinarian has made no progress in the present Congress, owing to the fact that, in the House, Speaker Reed has made no committee appointments. A new bill was introduced, but remains in this position, though the promise of its passage is very flattering should the opportunity arise.

*Tennessee.*—This State seems to be very much in the position of Illinois relative to proper recognition of the veterinary profession in the public positions of the State. The Legislature appropriated $2500 for veterinary sanitary work there, and the State decided to allow but $600 of this amount for the State veterinarian. This salary was such as to remove from the list of candidates for this position the late State Veterinarian, W. C. Rayen, but was subsequently accepted by Dr. Schiebler. It seems as though this amount would allow but a very small amount of work to be done by this State official. In the city of Nashville the City Council created the office of city meat inspector, to be elected by the city Board of Health. A non-graduate, Dr. Collins, has been selected for this place in the face of the application for the position by two well-known graduates—Drs. T. W. Scott and Joseph Plaskett—and it seems as though the appointment was made solely on a political basis, the good work to be done by such an official being a secondary consideration. Such results are often due to failure of veterinarians to unite in their work and to the lack of proper agitation of the duties that fall to the lot of such officers; hence the results that follow. We would advise the veterinarians of Tennessee to use their State Association so far as possible in correcting these conditions by better education of the public to the importance of these places and the necessity of better combination to insure good work.

*Colorado.*—The bill destined to regulate the practice of veterinary science and to provide for a State veterinary examining board failed of passage, owing to a wrangle that the Legislature got into over other measures. A bill was passed by the House and Senate, but vetoed by the Governor, in reference to the inspection of sheep, which would have caused a suspension of the present quarantine regulations concerning "scab." A bill compelling the examination and qualification of horseshoers before allowing them to continue business in the State was carried through and signed by the Governor, and now becomes a law. The appropriation for the State Veterinary Sanitary Board, along with other departments of the State Government, was scaled down one-third.

*Iowa.*—Governor Drake, of Iowa, sent a special message to the State Legislature, asking the passage of the law requiring State and county officers to coöperate with the Bureau of Animal Industry in stamping out hog-cholera. An experimental district will be established and thorough sanitary measures adopted.

*Great Britain.*—The British Government is considering a measure known as the Marks bill, whose object is to have all meats, both foreign and domestic, marked and sold for what they are; dealers in foreign meats to be registered. Americans products need fear no shrinkage in their sale from such a law.

*Minnesota.*—The Legislature dealt quite generously with the veterinary profession during the past winter. We were so fortunate as to secure a revision of the laws dealing with infectious diseases of animals, and an increase of $3000 annually for this work. This is especially gratifying in view of the stringent financial situation. The State Board of Health, veterinarians, and stockmen of the State united in this effort and put their estimate for the needed increase at the smallest amount for which they believed the work could be done in a satisfactory way, and the Legislature generously gave just what was asked.

The law was revised on four essential points. 1. We have secured a rewording of several sections that were generally misunderstood by parties to whom circulars were sent in the course of State Board of Health work. 2. The authority for quarantine and the quarantine regulations are made more extensive and specific. 3. The annual appropriation increased $3000. 4. Fines were reduced so as to read "not more than $100 nor less than $25," in place of the previous reading, "not less than $100 nor more than $500." Under the new law each day's continuance of offence constitutes a separate offence and entails a repetition of the penalty. This change in the fines was made in order that suit could be brought in a justice's court, and the fines would be so moderate that the courts would be more willing to impose them. (A copy of the law is sent herewith.) There are several points of this law worthy of note—*e.g.*, the reading in section 2: "Any person who knows of or has reason to suspect the existence of any contagious or infectious disease in any domestic animal shall forthwith give notice thereof," etc. Again, in section 8: "The State Board of Health, or any duly authorized agent thereof, may examine or cause to be examined, under oath, all persons believed to possess knowledge of material facts, etc." In section 9: "Any person violating any provision of this Act, or any rule or regulation made by the State Board of Health, or any local board, or any order made by any such board, under the authority thereof, shall be guilty of a misdemeanor, etc." Section 10 gives an owner full authority to post notices on his farm during the prevalence of any contagious disease (with especial reference to hog-cholera), forbidding persons to enter, etc., and makes it a misdemeanor, punishable by fine, for any person not authorized by the State or local board of health to violate such notice. This gives owners authority to issue private quarantine to protect their herds in case the local boards are negligent or for any reason fail to give desired protection.

We have also secured an entire revision of our former Veterinary Practice Act. In this we have gained several important points: First, the Board of Veterinary Examiners shall hereafter consist of graduates of legally authorized and recognized veterinary colleges. Second, no person may hereafter commence the practice of veterinary medicine in this State who has not graduated from a recognized veterinary college. A non-graduate cannot even appear for examination hereafter. The graduate must not only submit his diploma, but must also pass any examination that may be required by the State Board. This Act takes effect from and after its passage.

When the former bill was passed the first point mentioned was neglected, and as a result we have been in the strange position of having a non-graduate upon the Board of Veterinary Examiners —*i.e.*, a non-graduate to sit in judgment upon the diplomas and qualifications of graduates who make application for certificates.

